# The paraspinal muscle-tendon system: Its paradoxical anatomy

**DOI:** 10.1371/journal.pone.0214812

**Published:** 2019-04-08

**Authors:** Maud Creze, Marc Soubeyrand, Olivier Gagey

**Affiliations:** 1 Radiology Department, Bicêtre Hospital, APHP, Le Kremlin–Bicêtre, France; 2 Complexité, Innovations, Activités Motrices et Sportives, CIAMS (EA4532), Paris-Sud University, Paris-Saclay University, Orsay, France; 3 Imagerie par Résonance Magnétique Médicale et Multi-Modalités, IR4M, CNRS, Paris-Sud University, Paris-Saclay University, Orsay, France; 4 Orthopedics Department, Bicêtre Hospital, APHP, Le Kremlin–Bicêtre, France; Virginia Commonwealth University, UNITED STATES

## Abstract

Anatomy of the muscle-tendon system is an important component to musculoskeletal models. In particular, the cross-sectional area of belly (mCSA) and tendon (tCSA) provides information about the maximum force that a muscle may exert. The ratio of mCSA to tCSA (rCSA) demonstrates how muscle force is related to the ability to resist/transmit the force to bone. Previous anatomical studies of the lumbar paraspinal muscles (LPM) showed that their bellies have large mCSA suggesting that they are powerful muscles. Surprisingly, surgical experience shows that the tendons of the LPM are among the thinnest tendons of the body. We therefore hypothesized that traditional biomechanics of the LPM and the rCSA do not correspond for LPM. In 10 fresh-frozen old cadavers, we measured the mCSA, tCSA and rCSA of the LPM (multifidus and the erector spinae, i.e. the longissimus and the iliocostalis); then, we compared these data with those of one of the weakest muscles in the body, i.e. the extensor digitorum communis (EDC) chosen because it shares some common anatomical features with the LPM, in particular with the erector spinae. For instance, the EDC has a polyarticular course and presents long and thin effector tendons. Among the LPM, the longissimus has the greatest mean ACSA with 10.42 cm^2^ compared with 9.16 cm^2^ for the iliocostalis and 0.24 cm^2^ for the multifidus. Mean ACSA of the EDC was almost ten times smaller than those of erector spinae. Regarding the mean tCSA, the EDC was the largest one with 11.48 mm^2^ compared with 2.69 mm^2^ and 1.43 mm^2^ for the longissimus, 5.74 mm^2^ and 2.38 mm^2^for the iliocostalis and 5.28 mm^2^ and 4.96 mm^2^ for the multifidus. Mean rCSAs of the erector spinae were extremely small, ranged from 1/156 for the spinal attachment of the iliocostalis to 1/739 for the rib attachment of the longissimus that suggests that tendons are an unsuitable size to transmit the force to bone. Mean rCSA of the multifidus and the EDC were in the same range with rCSA = 1/5 and rCSA = 1/9 respectively. The rCSA of the multifidus was substantial, but its ACSA (1cm^2^) corresponds to low-power muscles. This paradoxical anatomy compels us to consider the biomechanics of the LPM in a different way from that of the classical “chord-like model”, i.e. the muscle belly creates a force that is applied to a bone piece through a tendon. The LPM have large contractile mass in a semi-rigid compartment inside which the pressure may increase. This result strengthens the hypothesis that high pressure and intrinsic stiffness of the LPM create two stiff bodies, closely attached to the spine thus ensuring its stabilization.

## Introduction

Stabilization of the spine requires numerous and powerful mechanisms involving a huge myofascial complex and an aponeurotic girdle surrounding the spine [1, 2].

The main lumbar paraspinal muscles (LPM) are arranged into three muscular columns (lateral, the iliocostalis; intermediate, the longissimus and medial, the multifidus) enclosed in a semi-rigid cylinder formed by i) the thoracolumbar fascia (TLF), ii) the anterior wall build from the transverses process and the ligaments and iii) the medial wall build from the spinous processes and the ligaments. This cylinder is known as the paraspinal muscular compartment (PMC) [3–10]. The erector spinae, i.e. the longissimus and the iliocostalis, run the length of the spine from the sacral to the thoracic region and is attached to each thoracic and lombar vertebra and on the dorsal aspect of the inferior ribs [10, 11]. The multifidus consists of a number of fleshy and tendinous fascicles, which are inserted on the spinous process of each vertebra and distally attached to the three or four vertebras below [7, 12]. The exact function of the LPM remains unclear because of their high number of bundles, the varying obliquity of the fibres, their polyarticular course and their short lever arm.

According to the current “chord-like model” (CLM), a muscle belly creates a force that is applied to a bone piece through a tendon. LPM have one “fixed proximal” attachment on the dorsal part of the pelvis and pull on “the free mobile distal” attachments on the spine and the ribs through tendons that we shall call the effector tendons that provide dorsal extension of the spine [11, 12]. Many biomechanical models have been proposed, but presently none comprehensively describes the stabilization of the lumbar spine [4, 5, 13, 14]. Nevertheless, a better understanding of this function is an important issue because of the high prevalence of low back pain and its social consequences [15, 16]. Cross-sectional imaging investigations demonstrated that low back pain (LBP) might be associated with structural changes of the LPM including decrease in cross-sectional area (CSA) and increase in fat content [17]. It is now well recognized that tolerance of low back pain depends a great deal on the CSA of the LPM.

To measure the maximal force of a muscle directly is difficult in living subjects, since many muscles are working simultaneously. Anatomical study of a muscle provides only indications regarding its maximal force. Many parameters are used to calculate the strength of muscles, such as the length of the muscle fibres, the mass, the CSA and the pennation angle of muscle bellies [18–20]. For the tendons, the mass, the length and the CSA (tCSA) are measured. However, the rather complex anatomy -i.e. the LPM are multiceps, multipenate and polyarticular—of LPM makes this classical approach extremely difficult.

Obviously, a powerful muscle should be connected to a thick tendon, since the latter has to transmit huge forces [21]. Given their large volume, LPM must be considered as powerful muscles [22, 23]. For this reason, effector tendons, on which the force is applied, should be adapted to such power and have a large CSA. The maximum stresses a tendon could support, can be estimated by considering the relative CSA of each tendon and of the muscle belly (rCSA) [19]. However, surgical experience suggests that there is an absence of thick tendons connected to LPM.

We therefore hypothesized that the tCSA of LPM does not correspond to the forces applied by the related muscle. To investigate this, we measured length, thickness and width of the muscle bellies and effector tendons of the LPM and calculated the ratio of mCSA to tCSA (rCSA) to demonstrate how muscle force was related to the ability to resist/transmit the force to bone. To illustrate our findings, we also compared these anatomical measurements in the muscle belly and tendons of the LPM with those of the extensor digitorum communis (EDC), chosen because the EDC shares some common anatomical features with the LPM: i) it has a polyarticular course, ii) it has long and thin effector tendons with a tCSA that could be similar to that of the LPM according to the study of Ruggiero et al., iii) it has an optimal rCSA, and iiii) it provide an extension movement [20].

## Materials and methods

### Gross anatomy

Ten fresh-frozen adult human cadavers (6 females, 4 males, mean age: 77 ± 10 years) were dissected. None of the cadavers revealed any evidence of previous surgical procedures, spine deformation or traumatic lesions of the lumbar region.

Procedures and measurements related to the cadavers were approved by local ethic commitee. The committee waived the need for informed consent. The body donor was not from a vulnerable population and the donor or next of kin provided written informed consent that was freely given.

Dissections were performed at *Ecole de Chirurgie (Assistance Publique des Hôpitaux de Paris)*.

#### Lumbar paraspinal muscles

The specimens were positioned in the prone position, with the arm placed along the body. A large skin incision from C7 to S3 was performed. After removal the skin and the subcutaneous fat, the TLF was totally exposed [11, 24]. After resection of the TLF and the spinalis, the longissimus and the iliocostalis were first examined individually, then severed and removed in order to study the multifidus. For each muscle, we studied the disposition and attachments of bundles and tendons on the right and left sides. Altogether, morphometrical data of 360 fleshy fascicles and 1276 tendinous fascicles were recorded.

#### Extensor digitorum communis

After finishing the data collection on the LPM, the cadavers were turned. The skin and the subcutaneous fat of the forearm were removed bilaterally. The extensor carpi radialis longus and the extensor carpi ulnaris were retracted to expose the EDC. We studied the disposition and attachments of tendons bilaterally, and then the EDC was removed. Morphometrical data of 20 bellies and 75 tendinous fascicles were recorded.

### Measurement

Length, thickness and width were measured at their largest point for muscle bellies and at their origin for effector tendons using a micrometer (Silverline, United Kingdom).

For each muscle belly and each tendon, the anatomical cross-sectional area (tCSA for tendons, ACSA for muscle bellies) was defined using the following formula: CSA = width x thickness. Volume (belly and tendon) was defined using the following formula: Volume = length x thickness x width. The rCSA was calculated as follow: ACSA/tCSA [19–21].

### Statistics

Descriptive statistics were used for the measured variables. Since some variables were not normally distributed, we used nonparametric tests. A Wilcoxon matched-paired signed-rank test was performed to detect sex difference and differences between the left and right sides for length, thickness and width of muscle bellies and tendons, and the results from both sides were pooled. *P*-values of <0.01 were considered statistically significant.

## Results

### Description of the LPM

#### TLF and erector spinae aponeurosis (ESA) (Fig 1)

The TLF was an irregular, thick and diamond-shaped dense connective tissue covering the LPM from the lumbosacral region to the spinous process of T7. The TLF was strongly attached to the spinous processes of T7 down to S1 medially, to the transverse processes of L1 to L5 laterally, and caudally, to the posterior part of the sacrum and to the iliac crest. The TLF was in continuity with the aponeuroses of the abdominal wall muscles and limb muscles. Both the TLF and the vertebra delimit an inextensible circumferential belt around the LPM that may promote an increase of stiffness and pressure during LPM contraction.

**Fig 1:**
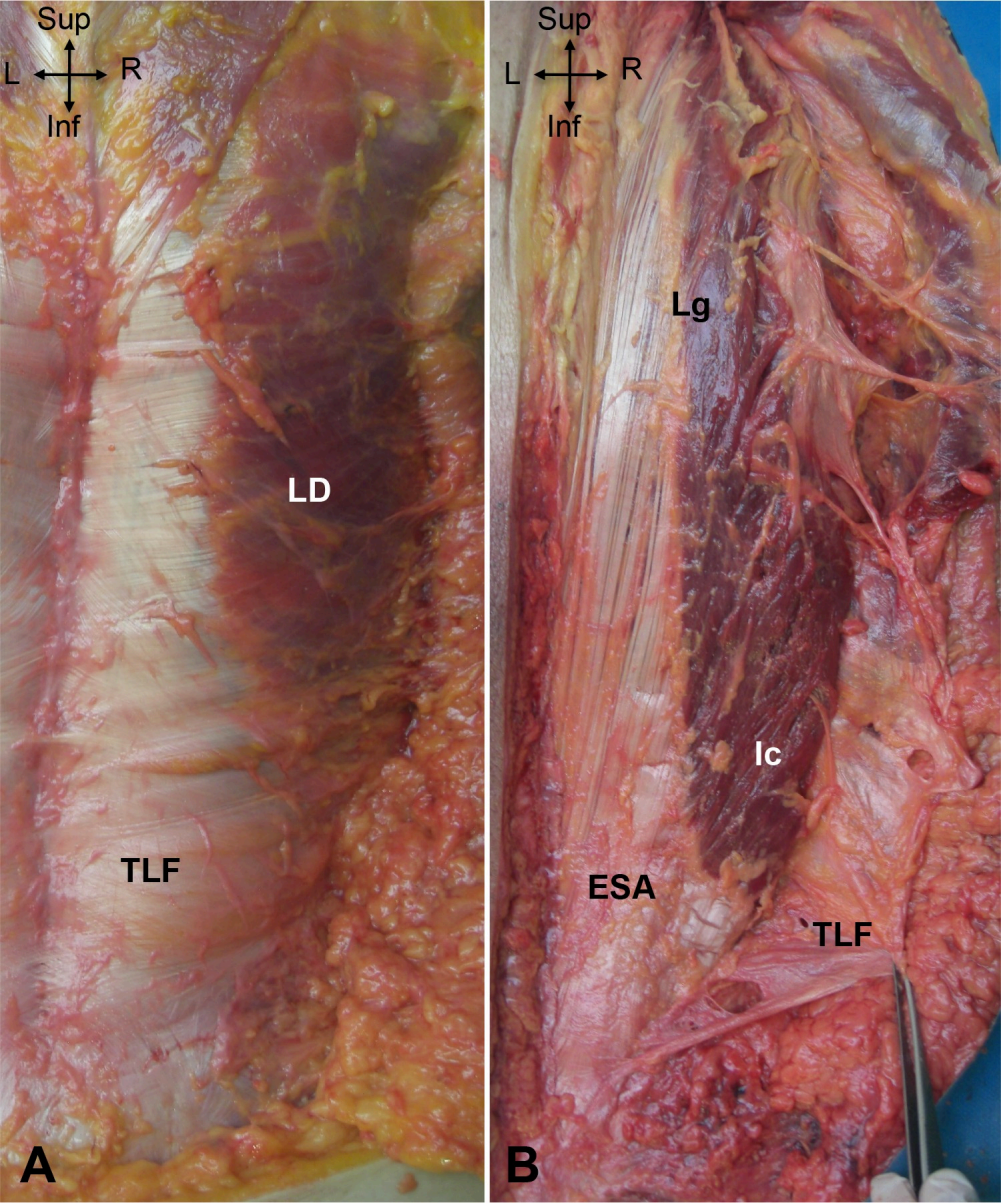
A. Posterior view of the thoracolumbar fascia (TLF) showing the common insertion with the latissimus dorsi (LD). B. Posterior view of the erector spinae aponeurosis (ESA), attached to the longissimus dorsi (Lg) and the iliocostalis (Ic). Inf: inferior; L: left; R: right; Sup: superior.

Situated beneath the TLF, the ESA resembled thick and regular dense connective tissue, extending from the posterior aspect of the sacrum (S3) and the iliac crest up to the thoracic region (T5). ESA and TLF attached at the same location on the sacrum, ilium and spinous processes. The ESA attached to the erector spinae along a large proportion of its length. Under L4-L5, the muscle belly of the ES progressively disappeared; thus, the ESA constituted the common tendon of the ES. At the lumbosacral level, the ESA covered the multifidus, which became dominant over the sacrum.

#### Erector spinae (Fig 2A)

The iliocostalis had four sites of attachments: on the spine, on the ribs, on the ESA and on the iliac crest. Spinal tendons were attached on the mammillary processes of L1 to L4. They ran almost horizontally from the medial part of the muscle belly. Rib attachments (N = 6) were on the angle of the ribs (R5 to R12), lateral to the attachments of the longissimus. They were all thin, but their width and length differed according the level of the rib attachment. Attachment on the seventh, eighth and ninth ribs were larger and shorter than those located above.

**Fig 2:**
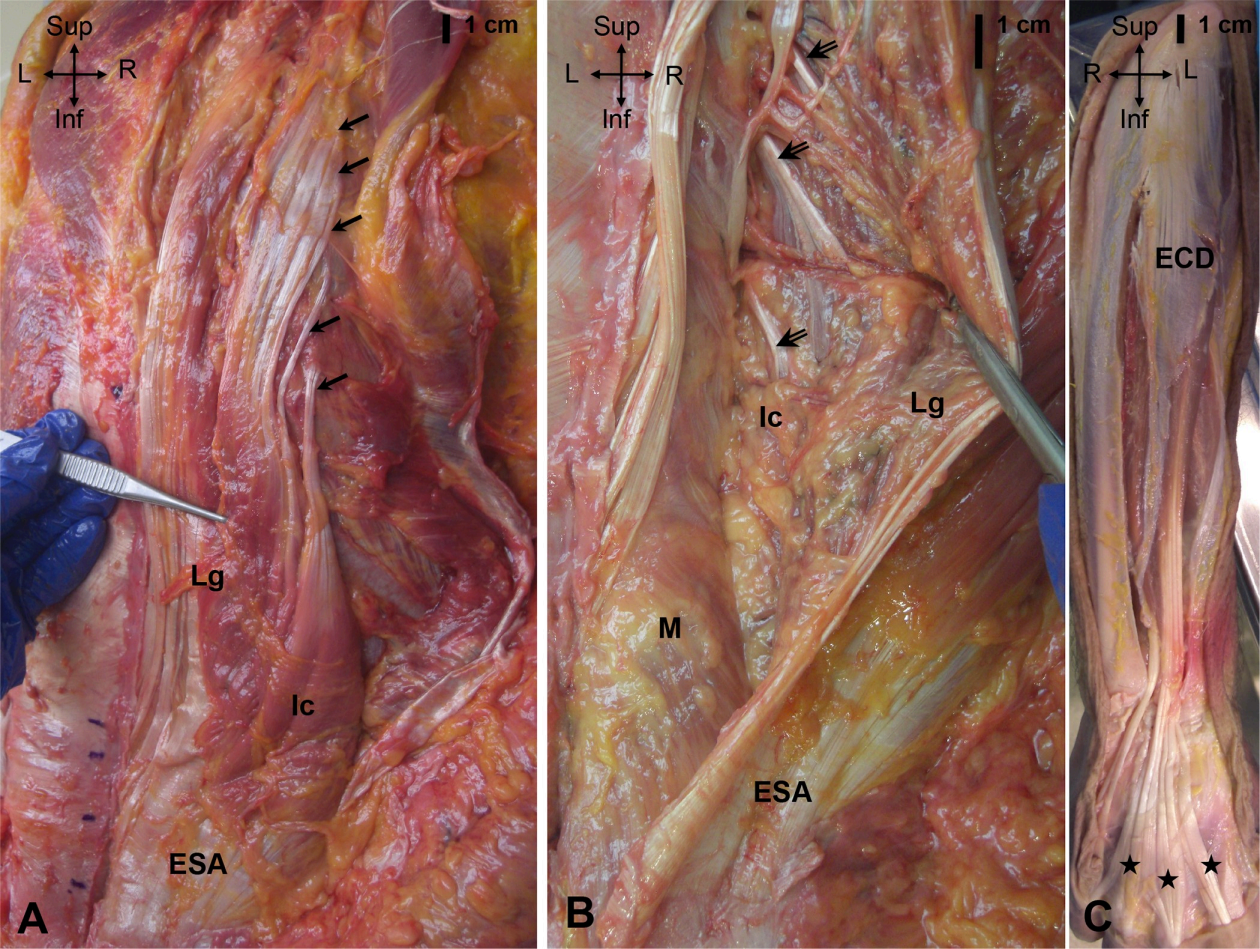
A. Posterior view of the longissimus dorsi (Lg). Arrows show the rib tendons of the iliocostalis (Ic). B. Posterior view of the multifidus (M); arrows show the spinal tendons of the longissimus. C. Posterior view of the extensor digitorum communis (EDC); stars show the digital attachments of the EDC. The longissimus had four sites of attachments: on the spine, on the rib, on the ESA and on the iliac crest. Spinal tendons (N = 7 to 8) were attached on the mammillary processes of the lumbar and thoracic vertebrae (T3-T4 to L5). They ran cranially and laterally from the inner part of the muscle belly. Rib attachments (N = 6 or 7) were located on the non-articular part of the tubercle of the ribs (R4 to R12), and were thin, long and almost transparent.

Note that, it was difficult to separate the belly of the spinalis from the belly of the longissimus. When the spinalis was distinguishable from the longissimus, it consists of a long and thin band of fibrous connective tissue with only few muscle fibers.

#### Multifidus (Fig 2B)

The cranial attachment was located on the spinous processes and caudal attachments on the mammillary processes of the three to four vertebras below, the sacrum and on the ESA. There was no tendon at the level of the sacrum, but there were aponeuroses as well as muscle fibres.

### Description of the extensor digitorum communis

The EDC arose from the lateral epicondyle of the humerus, from the intermuscular septa between it and the adjacent muscles, and from the antebrachial fascia. The EDC divided into four tendons at the middle of the forearm (Fig 2C). Then, the tendons diverged, passed on the back of wrist and the hand, and ended in the middle and distal phalanges of the fingers.

### Comparison of the two types of muscles

We did not found any significant side-related difference in the length (p = 0.21 for the longissimus, p = 0.43 for the iliocostalis, p = 0.07 for the multifidus, p = 0.60 for the EDC), width (p = 0.42 for the longissimus, p = 0.05 for the iliocostalis, p = 0.09 for the multifidus, p = 0.32 for the EDC), or thickness (p = 0.23 for the longissimus, p = 0.83 for the iliocostalis, p = 0.47 for the multifidus, p = 0.72 for the EDC) of the muscle belly as well as in the length (p = 0.34 for the longissimus, p = 0.04 for the iliocostalis, p = 0.87 for the multifidus, p = 0.07 for the EDC), width (p = 0.51 for the longissimus, p = 0.17 for the iliocostalis, p = 0.76 for the multifidus, p = 0.18 for the EDC), or thickness (p = 0.02 for the longissimus, p = 0.63 for the iliocostalis, p = 0.67 for the multifidus, p = 0.05 for the EDC) of the tendon. We did not found any significant sex-related difference in the length (p = 0.02 for the longissimus, p = 0.19 for the iliocostalis, p = 0.27 for the multifidus, p = 0.61 for the EDC), width (p = 0.23 for the longissimus, p = 0.28 for the iliocostalis, p = 0.47 for the multifidus, p = 0.54 for the EDC), or thickness (p = 0.09 for the longissimus, p = 0.92 for the iliocostalis, p = 0.16 for the multifidus, p = 0.04 for the EDC) of the muscle belly as well as in the length (p = 0.34 for the longissimus, p = 0.09 for the iliocostalis, p = 0.03 for the multifidus, p = 0.34 for the EDC), width (p = 0.88 for the longissimus, p = 0.24 for the iliocostalis, p = 0.55 for the multifidus, p = 0.38 for the EDC), or thickness (p = 0.92 for the longissimus, p = 0.72 for the iliocostalis, p = 0.42 for the multifidus, p = 0.02 for the EDC) of the tendon. Morphometric measurements and rCSA are summarized in Table 1.

**Table 1:** Cadavers anthropometric measures.

	Muscle belly (mean (standard deviation))					Tendon (mean (standard deviation))							
	Length (cm)	Thickness (cm)	Width (cm)	ACSA (cm^2^)	Volume (cm^3^)		Length (mm)	Thickness (mm)	Width (mm)	tCSA (mm^2^)	Ratio CSA	Volume (mm^3^)	Ratio volume
**Longissimus**	34.58 (5.60)	2.78 (0.61)	3.747 (0.94)	10.42 (3.16)	360.21 (33.55)	Spinal	28.65 (3.92)	0.65 (0.22)	4.14 (0.70)	2.69 (0.84)	1/387	77.07 (32.32)	1/4674
						Rib	36.00 (4.78)	0.32 (0.11)	4.49 (1.36)	1.43 (0.76)	1/739	51.72 (32.38)	1/6965
**Iliocostalis**	20.86 (4.15)	2.53 (0.70)	3.62 (1.22)	9.16 (0.85)	191.08 (19.82)	Spinal	19.23 (4.08)	0.82 (0.13)	7.01 (2.21)	5.74 (2.23)	1/156	110.53 (35.73)	1/1729
						Rib	44.14 (12.47)	0.59 (0.13)	4.04 (1.04)	2.38 (0.70)	1/385	105.21 (38.87)	1/1816
**Multifidus**	5.29 (0.77)	0.52 (0.16)	0.47 (0.09)	0.24 (0.09)	1.29 (0.43)	Spinous process	20.73 (5.60)	0.86 (0.19)	6.14 (1.79)	5.28 (2.37)	1/5	109.46 (47.62)	1/12
						Tansverse process	34.80 (4.04)	0.85 (0.12)	5.83 (2.37)	4.96 (1.98)	1/5	172.45 (78.00)	1/7
**Extensor digitorum communis**	14.71 (4.04)	0.82 (0.21)	1.23 (0.81)	1.01 (0.25)	14.84 (4.34)	Distal phalanx	184.57 (15.23)	1.27 (0.12)	9.04 (1.70)	11.48 (2.51)	1/9	2119.01 (110)	1/7

Length, thickness and width of the muscle belly of longissimus, iliocostalis, multifidus and extensor digitorum communis and length, thickness and width of the tendons of longissimus, iliocostalis, multifidus and extensor digitorum communis.

Among the LPM, the longissimus has the greatest mean ACSA with 10.42 cm^2^ compared with 9.16 cm^2^ for the iliocostalis and 0.24 cm^2^ for the multifidus. The ACSA of the EDC was 1.01 cm^2^.

Regarding the mean tCSA, the EDC was the largest one with 11.48 mm^2^ compared with 2.69 mm^2^ and 1.43 mm^2^ for the longissimus, 5.74 and 2.38 for the iliocostalis and 5.28 and 4.96 for the multifidus.

Mean rCSAs of the ES were extremely small, ranged from 1/156 for the spinal attachment of the iliocostalis to 1/739 for the rib attachment of the longissimus. Mean rCSA of the multifidus and the EDC were in the same range with rCSA = 1/5 and rCSA = 1/9 respectively.

## Discussion

In this anatomical study, we compared the powerful LPM with one of the weakest muscles in the body, i.e. the EDC, and demonstrated that the tCSA of the LPM were smaller than those of the EDC. We also found that the rCSA of the ES was extremely small.

There are very few published works about morphometic data of the LPM. Previous studies have measured the mACSA of the LPM at approximately 20 cm^2^ [9, 22]. This value corresponds to the mACSA of powerful muscles like the quadriceps, the latissimus dorsi or the triceps brachii. The LPM should produce a dorsal extension of the spine, with an exerted force of between 100 and 200 KN [20, 25–27].

Our study reveals that the effector tendons of the ES have a lower tCSA than EDC, which has among the thinnest tendons of the limbs and whose maximum force is estimated at 20N [20, 21, 28, 29]. The maximum stress that a tendon can support can be estimated by considering the rCSA [19]. In mammals, the optimal rCSA is estimated to be 1/34 for upper and lower limb muscles. The very small rCSA we found for ES suggests that the effector tendons are an unsuitable size to resist/transmit the force to bone. Contrariwise, the rCSA of the multifidus was high and could be able to support a high force applied on it, but its mACSA was less than 1cm^2^, which corresponds to low-power muscles like the extensor pollicis brevis [20, 29].

In a standing person, the lumbar spine sustains a heavy load; this has been estimated as being many hundreds of pounds. When bending forwards and picking up a heavy weight, the load may reach thousands of pounds [30]. Thin tendons, in particular tendons of the erector spinae, cannot transmit the required forces. Hence, the erector spinae cannot act as mobilisors of the spine, i.e. create significant joint movement, as suggested by previous authors and should be considered as stabilisor of the spine just like the multifidus [31, 32]. Moreover, so thin muscles such as the multifidus cannot provide adapted forces [33]. Even taking into account the limits of our study, the comparison between LPM and EDC is so strong that it should be taken into consideration.

Therefore, a paradox arises: there is a great discrepancy between the potential power of the LPM bellies and the size of their effector tendons. Through their effector tendons, the LPM cannot pull the thorax as well as the vertebrae strongly enough to provide direct spine extension from the full bending position. Consequently, the CLM is not able to accurately explain stabilization and mobilization of the spine. This suggests a more complex muscular strategy is required.

This study has limitations. Most studies dealing with ACSA and tCSA have been carried out in limbs, in which most muscles work according to the CLM. Thus, exact comparison with the LPM is not possible. The cadavers studied here were old. The comparison with a young specimen is obviously inapplicable due to the degenerative muscle and tendon changes that occur during aging. However, degenerative changes in the muscles are probably more pronounced than those of the tendons, thus the rCSA would be lower in younger adults. Our work does not evaluate the muscle force produced by the LPM. The complex anatomy of LPM makes it difficult to determine the physiological CSA (PCSA), which includes pennation angle, and indeed the force. We therefore used an indirect and simple method: comparing, in the same specimen, the LPM with a muscle whose effector tendons have the same tCSA as those of the LPM, which would allow a semi-quantitative comparison. Several methods exist to approximate muscle force [18]. Therefore, it is difficult to compare accurately the present results to those of studies that used different methods. It should be noted that the range of ACSA values remains the same regardless of the methods used [20, 34]. Taking into account the pennation angle, the PCSA would be superior to the ACSA, therefore the bias, i.e. that the ACSA likely overestimated the force of the LPM, in our study actually strengthens our results. Comparison between the LPM and the EDC is also a limitation since the EDC acts as a prime mover for finger extension while the LPM act as a fixator for the spine. Moreover, LPM are made of multiple layers of fleshy fascicles while the EDC had a single fusiform belly. But, surgical experience and previous anatomical studies showed that EDC has among the smallest tendons in the human body [20].

The paradoxical anatomy of the LPM raises two questions: why do the LPM have such volume when it appears that they cannot pull strongly on their tendons, and how can we explain the function of the muscles? LPM function needs to be discussed in terms of both spinal stabilization and spinal motions. Various hypotheses have been proposed to explain the remarkable myofascial stabilizing system of the spine; our findings provide anatomical arguments in favour of them [13, 35].

Stabilization of the lumbar spine, during walking for instance, requires isometric contraction of LPM. We hypothesize that the contraction of LPM does not act to pull on effector tendons but mainly to increase the stiffness of the PMC in order to provide spine stabilization. Biomechanical concepts related to the *hydraulic* and *viscoelastic* properties of the PMC have been proposed to describe the mode of action of the LPM. During standing postures, muscle contraction leads to an increase in muscle radius [3, 36–41]. When the LPM bulge, compartmentalization of the LPM by the TLF is responsible for a *hydraulic amplifier effect* that increases pressure within the PMC and thus increases stiffness of the spine [36, 42, 43]. As a consequence, the PMC acts as a posterolateral *bone-muscle composite beam*, which stiffens to stabilize the lumbosacral spine [13, 35].

In addition, the length of the LPM tendons and the huge dense connective tissue, i.e., the TLF and the ESA–which are among the thickest fascia in the body, allows storage of elastic strain energy [14, 19, 44, 45]. Ventilation, intra-abdominal pressure and co-activation of the psoas and abdominal wall muscles all provide stability during both standing posture and gait [2, 30, 46]. The ventral flexion of the trunk arises mainly though hip flexion; the lumbar spine alone has a low range of mobility. From full flexion of the trunk until the standing position, the main working muscles are the hip extensors, especially the gluteus maximus [47]. During this movement, energy storage from tendons, TLF and ESA might provide a recoil mechanism responsible for extension moments of the spine and the pelvis.

Beside strength and endurance, substantial ACSA appears essential to the properly functioning of the LPM, and more largely to the properly functioning of the PMC. LPM volume should be sufficient i) to fill up the PMC, ii) to provide enough pressure and stiffness within the PMC, iii) hence, to stabilize the spine. LBP is associated with LPM dysfunction [48]. It has been reported that LPM dysfunction lead to stiffness changes found by palpation, intramuscular pressure changes and changes in muscles size [49, 50]. Previous studies demonstrated that the core exercise–induced ACSA increase was responsible for decrease of the LBP [51–53]. Results of our study suggest it could be beneficial to look at rehabilitation techniques that favour the ACSA increase of both the multifidus and the erector spinae (and not only the multifidus, as promoted by some authors) in order to achieve an optimal stiffness of the PMC. Also, spinal surgical procedures should preserve the postoperative ACSA of the LPM [52, 53]. Hence, surgeons propose now minimally invasive procedure either anterior or oblique lumbar inter body fusion to spare the LPM [54].

Anatomy therefore provides strong arguments to change the functional paradigm of LPM. In other words, their function should be considered in a different way from the CLM. We have previously established that to understand the function of the deltoid muscle, the pressure applied by the muscle to the underlying upper end of the humerus should be taken in account [55]. The paradigm of a muscle pulling on its tendon is far from being sufficient to account for spinal stabilization and dorsal extension; it needs to be complemented by other properties such as 3D shape, volume variation, muscle perfusion and stiffness that together create mechanical interaction at the level of the bone-muscle interface. LPM are integrated to the cantilever spine system and should not be considered as external agents acting on the spine.
